# Mobile App to Streamline the Development of Wearable Sensor-Based Exercise Biofeedback Systems: System Development and Evaluation

**DOI:** 10.2196/rehab.7259

**Published:** 2017-08-21

**Authors:** Martin O'Reilly, Joe Duffin, Tomas Ward, Brian Caulfield

**Affiliations:** ^1^ Insight Centre for Data Analytics University College Dublin Belfield Ireland; ^2^ School of Public Health, Physiotherapy and Sports Science University College Dublin Dublin Ireland; ^3^ Biomedical Engineering Research Group Department of Electronic Engineering Maynooth University Maynooth Ireland; ^4^ Insight Centre for Data Analytics Maynooth University Maynooth Ireland

**Keywords:** exercise therapy, biomedical technology, lower extremity, physical therapy specialty

## Abstract

**Background:**

Biofeedback systems that use inertial measurement units (IMUs) have been shown recently to have the ability to objectively assess exercise technique. However, there are a number of challenges in developing such systems; vast amounts of IMU exercise datasets must be collected and manually labeled for each exercise variation, and naturally occurring technique deviations may not be well detected. One method of combatting these issues is through the development of personalized exercise technique classifiers.

**Objective:**

We aimed to create a tablet app for physiotherapists and personal trainers that would automate the development of personalized multiple and single IMU-based exercise biofeedback systems for their clients. We also sought to complete a preliminary investigation of the accuracy of such individualized systems in a real-world evaluation.

**Methods:**

A tablet app was developed that automates the key steps in exercise technique classifier creation through synchronizing video and IMU data collection, automatic signal processing, data segmentation, data labeling of segmented videos by an exercise professional, automatic feature computation, and classifier creation. Using a personalized single IMU-based classification system, 15 volunteers (12 males, 3 females, age: 23.8 [standard deviation, SD 1.8] years, height: 1.79 [SD 0.07] m, body mass: 78.4 [SD 9.6] kg) then completed 4 lower limb compound exercises. The real-world accuracy of the systems was evaluated.

**Results:**

The tablet app successfully automated the process of creating individualized exercise biofeedback systems. The personalized systems achieved 89.50% (1074/1200) accuracy, with 90.00% (540/600) sensitivity and 89.00% (534/600) specificity for assessing aberrant and acceptable technique with a single IMU positioned on the left thigh.

**Conclusions:**

A tablet app was developed that automates the process required to create a personalized exercise technique classification system. This tool can be applied to any cyclical, repetitive exercise. The personalized classification model displayed excellent system accuracy even when assessing acute deviations in compound exercises with a single IMU.

## Introduction

### Background

Exercise rehabilitation for the treatment of musculoskeletal conditions such as osteoarthritis, following an injury or orthopedic surgical procedures, is accepted as an essential treatment tool [1-3]. Resistance training may also be used to improve one’s muscular strength, hypertrophy, and power in nonpatient populations [4-6]. However, many people completing exercise programs encounter a variety of difficulties when performing their exercises without the supervision of a trained exercise professional such as a physiotherapist or strength and conditioning (S&C) coach. One such difficulty is that in many circumstances, people may execute their exercises incorrectly [7,8]. Incorrect alignment during exercise, incorrect speed of movement, and poor quality of movement may have an impact on the efficacy of exercise and may therefore result in a poor outcome [7,8]. It is therefore essential that accurate assessment of exercise performance is available to ensure that people perform their exercises properly. This is particularly necessary in cases where an individual completes their exercise program in the absence of an exercise professional’s supervision, for example, during home-based rehabilitation programs or S&C programs where the person performing the exercises cannot afford a personal trainer.

Recent research has shown inertial measurement unit (IMU)–based biomechanical biofeedback systems to be an accurate exercise assessment tool. Biomechanical biofeedback involves (1) the measurement of one’s movement, postural control, or force output and (2) the provision of feedback to the user regarding these measurements [9]. IMUs are able to acquire data pertaining to the linear and angular motion of individual limb segments and the center of mass of the body. They are small, inexpensive, and easy to set up, and facilitate the acquisition of human movement data in unconstrained environments [10]. Research in this field has shown the ability of multiple body-worn IMUs to evaluate exercise quality for a variety of exercises [11-14]. These range from early-stage rehabilitation exercises such as heel slides and straight leg raises [15] to more complex late-stage rehabilitation exercises or S&C exercises such as bodyweight squats [16], lunges [17], and single-leg squats [18-20]. More cost-effective and practical systems using a single body-worn IMU have also been shown to be effective in the analysis of exercise technique [17,18,21,22]. Systems that are based on a single IMU are considered preferential, as they can provide equivalent exercise analysis quality to multiple IMU setups at a lower cost.

However, in a number of cases, a single IMU setup achieves lower quality exercise analysis levels than multiple IMU setups. The ability of a single IMU setup to detect acute naturally occurring technique deviations in compound late-stage rehabilitation and S&C exercises such as deadlifts, lunges, and squats is also largely unknown; although this has been shown as possible for single-leg squats [18], the reported findings on lunges and squats pertain to detecting deliberately induced exercise technique deviations [16,17]. There is also a need to iteratively improve the accuracy, sensitivity, and specificity of IMU-based exercise technique biofeedback systems and increase the number of exercises that can be analyzed with IMUs. IMU-based exercise biofeedback systems should be able to assess technique for a comprehensive range of exercises, both accurately and in a manner that is practical for people completing the exercises.

There are a number of considerable challenges in the creation of such biofeedback systems. First, for machine learning classification algorithms to produce desirable results, they require large volumes of training data. As such, it is difficult to collect IMU data on a large variety of exercises in a research environment. Subsequently, current research has mainly assessed very commonly completed exercises that span the scope of musculoskeletal screening, rehabilitation, and S&C. There remain thousands of exercises for which the ability of IMUs to assess their technique is unknown. Classification algorithms such as random forests and logistic regression also require balanced training datasets, where each class (eg, acceptable or aberrant) has the same amount of instances in the training data [23-25]. This provides a huge challenge in creating systems that aim to detect natural technique deviations that occur idiosyncratically and at greatly differing frequencies. This challenge is heightened in circumstances where the intersubject variation of completing an exercise with acceptable form exceeds the intrasubject variation between one’s acceptable and aberrant form.

One solution to combatting the aforementioned challenges may be to create individualized exercise classification systems. In this circumstance, a classifier is created using training data solely from the person whose exercise is to be assessed. Preliminary research has shown that such classifiers can produce superior accuracy as compared with global classification systems [26,27]. Additionally, some global classification systems have only been developed and evaluated with deliberately induced technique deviations [16,17]. Personalized systems may allow for many more exercises to be evaluated for a particular person performing the exercises and could allow for acute naturally occurring technique deviations to be detected with a single body-worn IMU where this has not been previously possible. The classifiers would also be less memory intensive and more efficient, as they are developed using smaller training datasets. However, to the best of the authors’ knowledge, there is a lack of tools currently available to efficiently capture and label IMU data during exercise to enable the efficient development of personalized exercise technique classification systems.

### Objectives

Therefore, the purpose of this investigation was to create a tablet app that enabled efficient creation of personalized single IMU-based exercise biofeedback systems. We also sought to investigate the accuracy of this personalized system in a real-world evaluation using a sample of 4 compound lower limb exercises (lunges, single-leg squats, squats and deadlifts) in 15 participants. In this paper, an overview of the developed app is first presented. An experimental evaluation of the system in the real world is then described.

## Methods

### System Overview

**Figure 1:**
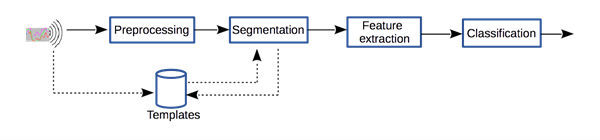
Steps involved in the development of an inertial measurement unit (IMU)–based exercise classification system.

In exercise classification with IMUs, there exist a number of universal steps that allow for the development of exercise biofeedback systems [28]. First, IMU data must be collected from participants as they exercise. Each repetition of each exercise must be labeled by an exercise professional. The signals collected from the IMU must be filtered to eliminate unwanted noise, and additional signals may be computed that, for instance, describe the IMU’s three-dimensional (3D) orientation. The signals are segmented into epochs, each of which pertains to one repetition of an exercise. Features are computed from these segmented signals as described in the upcoming “Feature Computation and Classifier Creation” section. Finally, a classification model is trained using both the labels provided by an exercise professional and the features computed from the sensor signals that pertain to the same repetitions (Figure 1). The tablet app, presented in this paper, allows for simultaneous IMU and video data capture. It then allows labeling of each IMU data epoch through reviewing its associated video epoch. Features are then automatically computed from the IMU signal epochs, and classifiers are built using these features and the labels provided by the exercise professional.

#### Overview of Data Collection Tool

**Figure 2:**
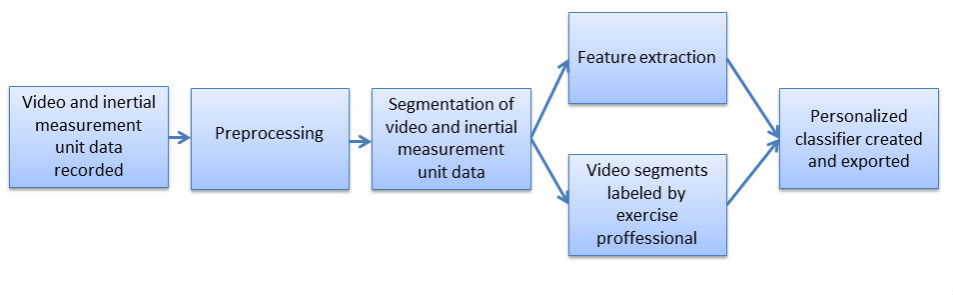
Schematic demonstrating the flow and functionality of the tablet app.

**Figure 3:**
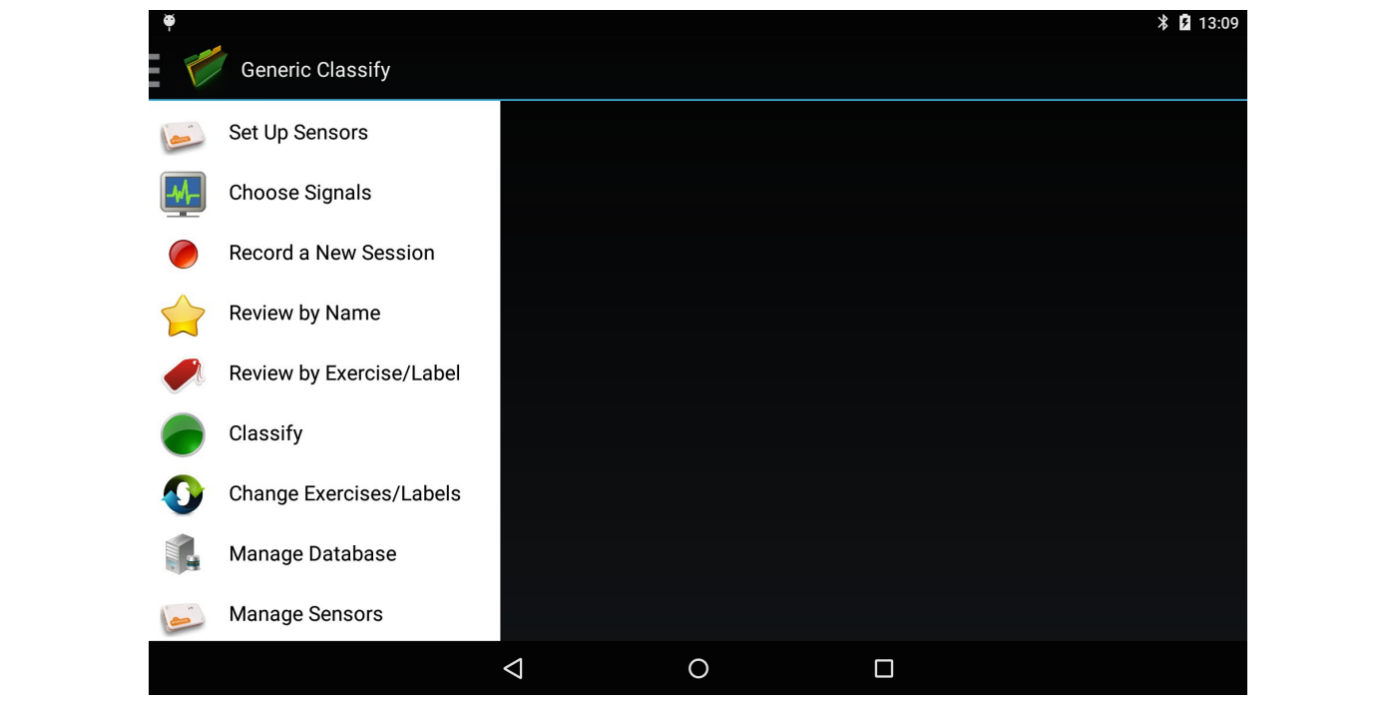
Home screen of tablet app, demonstrating its variety of functions.

The tablet app was developed using Android Studio (Android, Google) and ran on a Samsung Galaxy S2 tablet. It contains a number of tabs that enable a vast degree of functionality to enable the automated creation of personalized classification systems. Figure 2 demonstrates the processes involved and highlights the need for data labeling from an exercise professional. The various tabs within the app are demonstrated in Figure 3. The system can connect to a maximum of 5 Shimmer (Shimmer sensing) IMUs [29] and stream synchronized data from them simultaneously. All IMUs were automatically configured to stream triaxial accelerometer (±2 g), gyroscope (±500 ^°^/s), and magnetometer (±1.9 Ga) data at 51.2 Hz. These values were chosen, as they have previously been shown to be appropriate for the analysis of rehabilitation exercise with IMUs [15,18,19]. However, the sampling rate and sensor ranges may be insufficient for faster exercises such as jumping or plyometric exercises. Future iterations of the system will address this by allowing the exercise professional to select sampling rate and sensor ranges based on exercise type before data collection. For this study, the IMU was calibrated by the lead investigator of this study. This took roughly 10 min.

The app then allows for the automation of all the aforementioned steps in the development of an exercise technique classifications system as shown in Figures 1 and 2.

#### Video and IMU Data Collection

**Figure 4:**
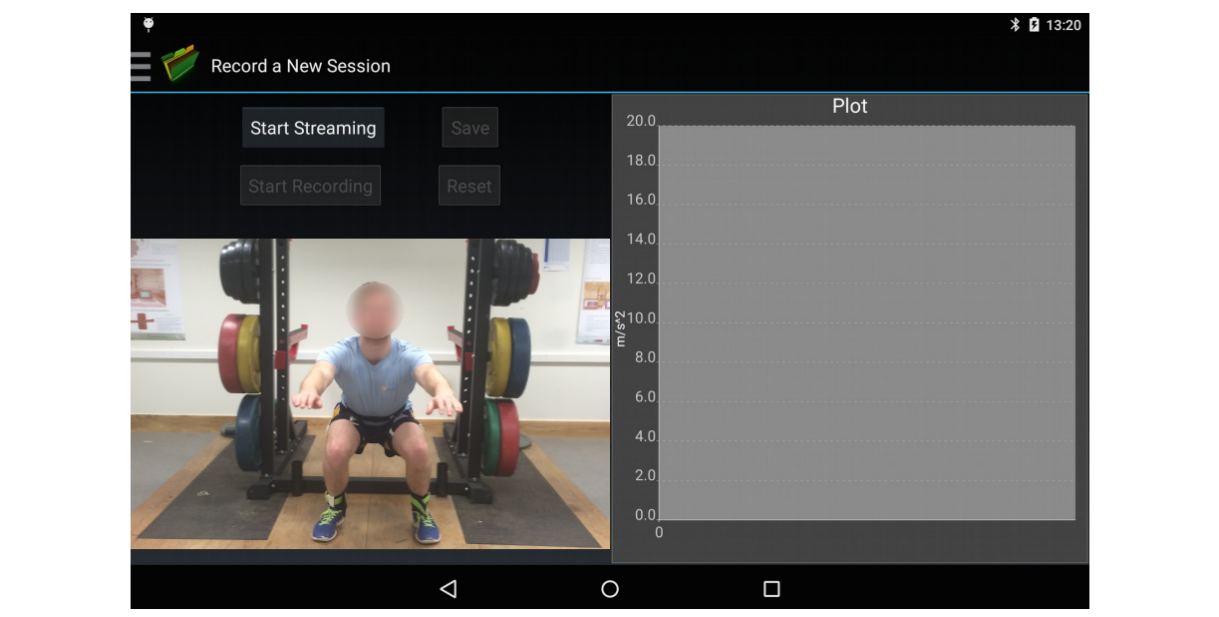
Data capture part of the app that allows IMU (inertial measurement units) data and video to be captured simultaneously.

Following sensor set up, navigating to the “Record a New Session” tab allows an exercise professional to take a video of their client as they exercise, as data from the IMUs are simultaneously collected. The video is captured at the tablet’s natural sampling rate, and IMU data are collected at 51.2 Hz (Figure 4). The exercise professional may choose to record their client from the frontal or sagittal plane depending on the exercise being evaluated.

#### Signal Processing and Segmentation

Following the recording of a set of a particular exercise, a number of steps were completed by the app in processing the IMU data. To ensure that the data analyzed applied to each participant’s movement and to eliminate unwanted high-frequency noise, 6 signals were low-pass filtered at f_c_=20 Hz using a Butterworth filter of order n=8. Nine additional signals were then calculated. The 3D orientation of the IMU was computed using the gradient descent algorithm developed by Madgwick et al [30]. The resulting quaternion values (W, X, Y, and Z) were then converted to pitch, roll, and yaw signals. The pitch, roll, and yaw signals describe the inclination, measured in radians, of each IMU in the sagittal, frontal, and transverse planes, respectively. The magnitude of acceleration was also computed using the vector magnitude of accelerometer *x, y,* and *z*. The magnitude of acceleration describes the total acceleration of the IMU in any direction. This is the sum of the magnitude of inertial acceleration of the IMU and acceleration due to gravity. Additionally, the magnitude of rotational velocity was computed using the vector magnitude of gyroscope *x*, *y*, and *z*. Although these magnitude signals do not allow for specific body segment planes to be analyzed, they can aid in capturing detection of aberrant movement when deviations are very pronounced or occur in multiple planes.

**Figure 5:**
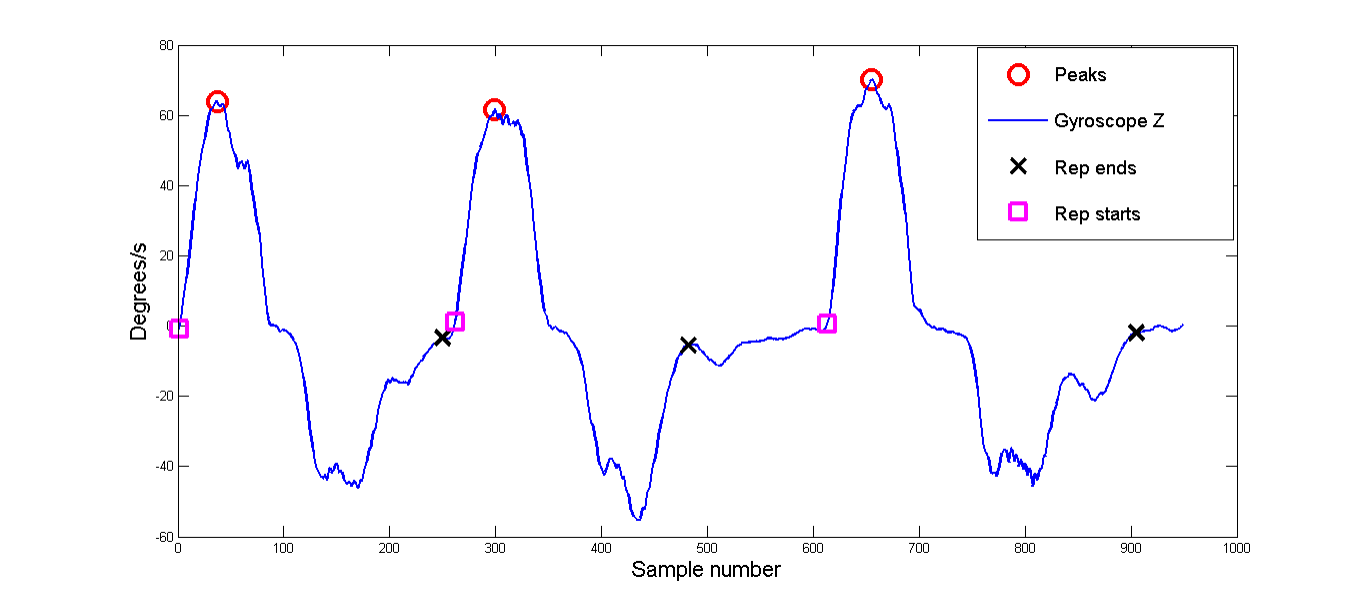
Plot showing detection of peak, start, and end points of repetitions through identifying neighboring zero-crossing values to the peak locations. The signal shown is the gyroscope Z signal from the left thigh during 3 repetitions of a deadlift.

The signals and video data were then programmatically segmented into epochs that relate to single full repetitions of the completed exercises. Many algorithms are available to segment human motion for rehabilitation exercises, including the sliding window algorithm [31]; top-down, bottom-up algorithms [32]; zero velocity–crossing algorithms; template-base matching methods [33]; and the combination algorithms of the above [34]. These algorithms have advantages and disadvantages. For the purpose of the creation of a functioning classifier creation tool, a simple peak-detection algorithm was used on the gyroscope signal with the largest amplitude for any particular exercise. The start and end points of each repetition can then be found by looking for the corresponding zero-crossing points of the gyroscope signal leading up to and following the location of a peak in the signal. Figure 5 demonstrates example results of the segmentation algorithm used on the gyroscope Z signal, from an IMU positioned on the left thigh during 3 repetitions of the deadlift exercise.

Following the signal processing and segmentation of the IMU data, the video was cut into epochs based on the start and end points of repetitions found in the IMU data. The session name, exercise name, repetition number, IMU data, and video data for each individual exercise repetition were stored as objects in a database.

The specific signal processing and segmentation processes selected were chosen based on their demonstrated capability in similar research [16-19]. In future iterations of the app, a variety of additional signal processing and segmentation options may be presented to the exercise professionals using the system, or the functions will be updated to match the emerging state of the art.

#### Data Labeling

The app enables a number of different functionalities regarding data labeling. The exercise professional using the tablet app first has the ability to add new exercises and technique deviations as possible labels for the stored and segmented data. These labels also become available to the exercise professional when they record new exercise sessions.

The exercise professional then has the option of labeling the videos, repetition-by-repetition, through viewing them according to the filter criteria “session name” or by “exercise type.” The default class for all repetitions is “Acceptable” until they are labeled as “Aberrant” or as a specific deviation from an acceptable technique. An unlimited number of possible labels can be created for each exercise.

**Figure 6:**
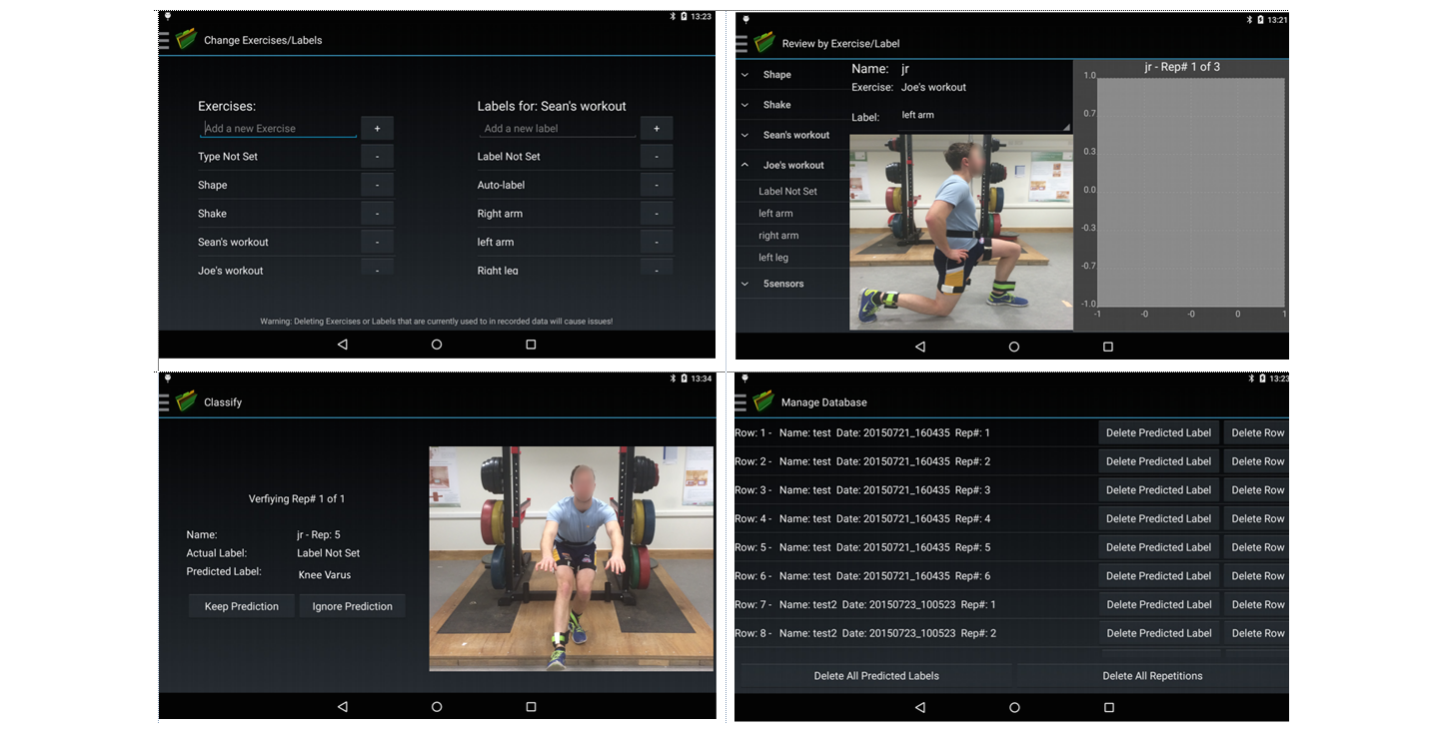
Various data labeling functionalities of the app.

Once data have been collected for each exercise, there is also an “Auto-label” function. This function uses data already labeled by the exercise professional to build a random forests classifier, which estimates the class for currently unlabeled data. As shown in Figure 6, the app then presents the classifier’s predicted label with the video of the repetition and allows the exercise professional to either keep the prediction or ignore the prediction. If the prediction is ignored, the repetition can then manually be labeled in the “review by exercise” or “review by session” tab. The database can also be manually updated at any time, allowing the exercise professional to remove particular repetitions or edit the current label for it. Figure 6 highlights the app’s various data-labeling functionalities.

#### Feature Computation and Classifier Creation

Once the data have been labeled as desired by the exercise professional, the app can then build the personalized exercise technique classification objects for each client and each exercise they completed. A separate classifier is created for each different exercise.

Time-domain and frequency-domain descriptive features are computed to describe the pattern of each of the 18 signals when the 5 different exercises were completed. These features were, namely, “Mean,” “RMS,” “Standard Deviation,” “Kurtosis,” “Median,” “Skewness,” “Range,” “Variance,” “Max,” “Min,” “Energy,” “25th Percentile,” “75th Percentile,” “Level Crossing Rate,” “Fractal Dimension” [35], and the “variance of both the approximate and detailed wavelet coefficients using the Daubechies 5 mother wavelet to level 7.” This resulted in 17 features for each of the 18 available signals, producing a total of 306 features per IMU. Training data are balanced to ensure the developed classifiers are unbiased. This is done by removing random observations of overrepresented classes until all classes have an equal number of observations. For instance, if a labeled dataset of squat repetitions has 50 “acceptable” repetitions and 40 “aberrant” repetitions, 10 “acceptable” repetitions, which are chosen randomly using a programmatic method, will not be used to train the classifier. Finally, the app builds random forests classifier objects with 400 trees.

The choice of features computed, balancing of training data, and use of a random forests classifier all replicate recently published work in the field [15-18]. Similar to signal processing and segmentation, these processes can be updated in future iterations of the app to match the emerging state of the art in exercise technique classification with IMUs.

The developed classifier objects can then be exported from within the tablet app to individual’s exercise biofeedback apps on their mobile phones for use in monitoring their rehabilitation exercise programs.

### System Evaluation

#### Participants

Fifteen volunteers currently not undergoing any rehabilitation participated, whereby no participant had a current or recent musculoskeletal injury that would impair their exercise performance. Participants were recruited via poster advertisements on notice boards in the local area and were, therefore, a sample of convenience. Of these, 5 participants were beginner exercisers who had been screened to have naturally aberrant technique and were untrained in the exercises in the study, whereas 10 participants were experienced with the exercises and were required to deliberately mimic aberrant technique at appropriate times during the experiment. Each participant signed a consent form before completing the study. The University College Dublin Human Research Ethics Committee approved the study protocol.

#### Experimental Protocol

The testing protocol was explained to the participants upon their arrival at the research laboratory. Their gender was recorded and their weight was measured using a weighing scale. Height was then measured with a stadiometer. All participants completed a 5-min warm-up on an exercise bike, during which they were required to maintain a power output of 100W and cadence of 75 to 85 revolutions per minute. Following the warm-up, an investigator placed a single IMU on the participant at the midpoint of the left femur (determined as halfway between the greater trochanter and lateral femoral condyle). The orientation and location of the IMU was consistent across all study participants. The IMU sampling rate and sensor range settings used were identical to those described in the “Overview of tool” section.

Video and IMU data were then simultaneously collected as the participant completed 4 of the following exercises: bodyweight left leg, single-leg squats; bodyweight lunges; bodyweight or barbell squats; and barbell deadlifts. These exercises were chosen pragmatically, as they represent compound lower limb exercises that span both the late-stage rehabilitation (knee, kip, and ankles) and S&C domains. They also cannot be easily analyzed by any existing systems. Forty repetitions of each exercise were collected; 20 repetitions were completed with “acceptable” form, whereas 20 repetitions were completed with “aberrant” form. The “aberrant” repetitions from the 5 beginners were naturally occurring, whereas the 10 experienced participants deliberately induced their “aberrant” form. Following these data collection, the IMU was removed from the participants’ left thigh.

**Figure 7:**
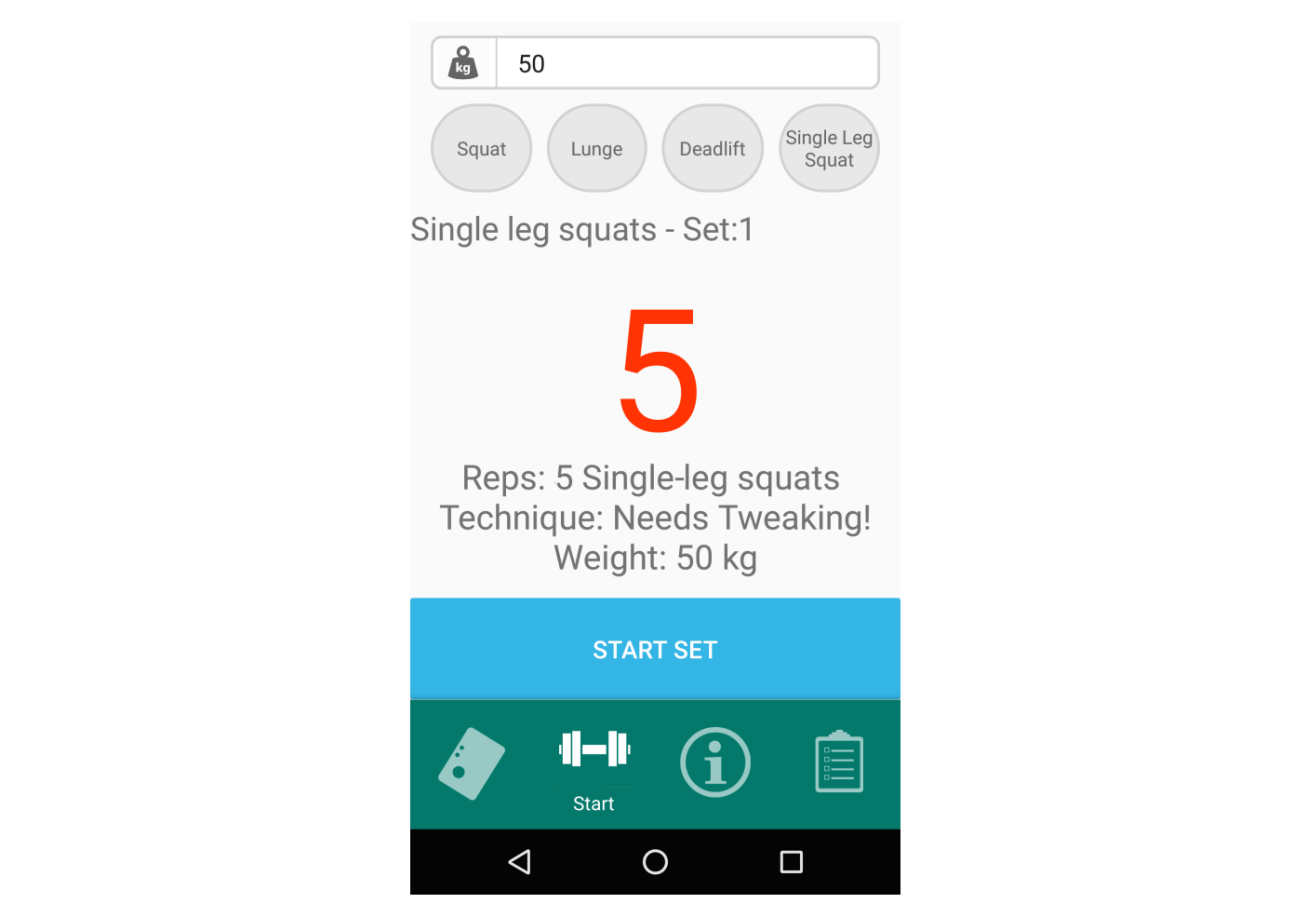
Screenshot from the “Formulift app,” which uses the classifiers developed from the tablet app to analyze whether a person’s exercise technique is acceptable or aberrant as they complete squats, deadlifts, lunges, and single-leg squats.

As the participant rested, the exercise professional then used the segmented videos to label all exercise repetitions of the 4 exercises as being “acceptable” or “aberrant” technique (160 repetitions per participant). For each participant, 4 binary random forests classifiers were then created, each pertaining to 1 of the 4 aforementioned exercises. These random forests objects were imported into a biofeedback app. The data labeling and classifier creation took a maximum of 30 min per participant. The biofeedback app entitled “Formulift” (Figure 7) allows a person performing the exercises to connect to a Shimmer IMU, select each of the above exercises, and have their repetitions of each exercise be classified as “acceptable” or “aberrant.”

Following the creation of their personalized biofeedback system, the participants first secured the IMU to their left thigh by themselves and connected the wireless Shimmer IMU to the mobile app. These steps took roughly 1 min. They then completed 2 sets of 10 repetitions for each of the 4 exercises. In the first set of each exercise, they were instructed to exercise with their best possible technique, and in the second, they were asked to try and replicate the mistake they had made before being coached by the exercise professional. The video of the whole session was simultaneously taken, and the classifier’s predictions of the participants’ technique were stored in the background storage folders on the tablet.

#### Data Analyses

Following the participants’ use of their personalized biofeedback app, the system’s predicted labels (acceptable or aberrant) for each repetition of each exercise were stored. The videos of each repetition of each exercise were then labeled by an S&C coach with more than 5 years’ experience in visual analysis of the exercises. They were labeled as acceptable or aberrant in a systematic format. The S&C coach could view the repetitions as many times as necessary to make a clear judgment on the label. Labeling all data for each beginner participant took under 25 min and was quicker for the experienced participants as their aberrant form was deliberately induced. Example types of aberrant form that the exercise professional was looking for included knee valgus, knee varus, and asymmetry as used in similar recent research [16-19].

**Figure 8:**
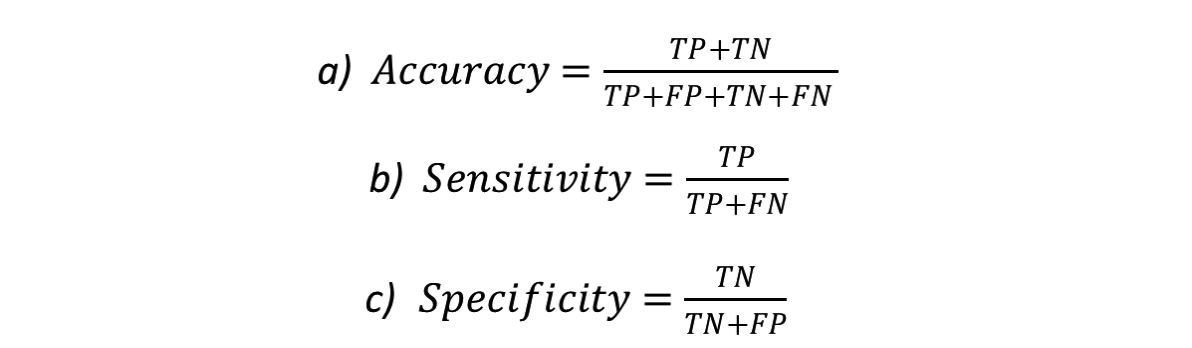
Formulae for: a) accuracy, b) sensitivity, and c) specificity.

The personalized, classifiers-predicted labels were then compared with the exercise professional’s labels, which were considered to be ground truth for each repetition of each exercise from each participant. Where the exercise professional had labeled a repetition as “acceptable” and the classifier predicted “acceptable,” this was counted as a true positive (TP). However, if the classifier predicted “aberrant,” in this circumstance a false negative (FN) was counted. If the exercise professional and classifier both deemed a repetition to be “aberrant,” it was counted as a true negative (TN). However, if the exercise professional deemed a repetition to be “aberrant” and the classifier predicted it as “acceptable,” this was counted as a false positive (FP).The scores used to measure the quality of classification were total accuracy, sensitivity, and specificity. Accuracy is the number of correctly classified repetitions of all the exercises divided by the total number of repetitions completed. This is calculated as the sum of the TPs and TNs divided by the sum of the TPs, FPs, TNs, and FNs. Sensitivity measures the effectiveness of a classifier at identifying a desired label, whereas specificity measures the classifier’s ability to detect negative labels. These three metrics were used to assess the classification quality of each individual participant for each of the 4 exercises completed. The formulae for accuracy, sensitivity and specificity are shown in Figure 8.

## Results

### Participant Demographics

The demographics of the participants were as follows: 12 males, 3 females, age: 23.8 [standard deviation, SD 1.8] years, height: 1.79 [SD 0.07] m, body mass: 78.4 [SD 9.6] kg. Each participant’s characteristics are shown in Table 1.

**Table 1:** Participant characteristics.

Type	Gender	Age, in years	Height, in meters	Weight, in kilograms
Beginner	Male	20	1.68	66.5
Beginner	Male	25	1.75	68
Beginner	Male	22	1.76	76
Beginner	Female	26	1.74	86
Beginner	Female	26	1.7	65
Experienced	Male	23	1.85	85
Experienced	Female	21	1.77	72.5
Experienced	Male	24	1.88	86
Experienced	Male	25	1.83	74
Experienced	Male	26	1.7	63
Experienced	Male	23	1.75	83
Experienced	Male	25	1.805	84
Experienced	Male	22	1.93	86
Experienced	Male	24	1.775	84
Experienced	Male	25	1.88	97
Mean (SD^a^)		23.8 (1.8)	1.79 (0.07)	78.4 (9.6)

^a^SD: standard deviation.

### System Evaluation Results

Table 2 demonstrates the mean accuracy, sensitivity, and specificity scores for all participants using their 4 personalized classifiers for each exercise under study, in the real-world evaluation as described in the “System Evaluation” section. The mean results for the 5 beginner participants who had naturally aberrant technique and for the more experienced participants who had deliberately induced technique mistakes are shown.

The system was more accurate for the experienced exercisers’ group (98.59%) than the beginners’ group (88.00%) for the deadlift exercise but was otherwise more accurate for the beginners. This is particularly interesting as the beginner’s technique aberrations were naturally occurring, and the experienced group’s aberrations were deliberately induced. The system was least accurate for lunges (84.14%) and most accurate for single-leg squats (97.26%) across all participants. Accuracy varied considerably for each individual in the lunge and squat exercises, as can be seen in the presented standard deviations (Table 2). The range of accuracies across all participants was less variable for the single-leg squat and deadlift exercises.

For the single-leg squat exercise, the mean sensitivity was 98% and the mean specificity was 93%. This means the system was better at detecting acceptable single-leg squat technique than aberrant technique or that 7% of aberrant exercise repetitions were misclassified as acceptable. The system had relatively similar sensitivity and specificity in classifying lunges and deadlifts. Therefore, it would not appear biased to either the “acceptable” or “aberrant” class to an exerciser using the system. However, for the squat exercise there was a 13% chance that an acceptable repetition may be classified as aberrant and a 17% chance that an aberrant repetition may be classified as acceptable.

**Table 2:** Mean accuracy, sensitivity, and specificity of personalized classifiers for the binary evaluation (acceptable or aberrant technique) of each exercise and each participant.

Exercise	Participants	Accuracy, mean (SD^a^), %	Sensitivity, mean (SD), %	Specificity, mean (SD), %
Single leg squats				
	Beginners (N=5)	99.17 (1.86)	100.00 (0.00)	98.33 (3.73)
	Experienced (N=10)	95.98 (6.69)	97.00 (4.83)	90.41 (15.24)
	All (N=15)	97.26 (5.54)	98.00 (4.00)	93.03 (19.09)
Lunges				
	Beginners (N=5)	92.63 (10.5)	96.67 (7.45)	88.70 (16.36)
	Experienced (N=10)	77.77 (21.26)	74.07 (3.19)	83.82 (32.17)
	All (N=15)	84. 14 (18.96)	83.11 (27.49)	85.78 (20.85)
Squats				
	Beginners (N=5)	84.83 (16.58)	75.00 (35.47)	95.00 (5.00)
	Experienced (N=10)	82.71 (15.43)	90.98 (15.25)	74.44 (32.01)
	All (N=15)	84.53 (16.38)	87.06 (27.53)	82.67 (29.00)
Deadlifts				
	Beginners (N=5)	88.00 (8.16)	84.00 (16.25)	90.00 (2.00)
	Experienced (N=10)	98.59 (2.71)	98.15 (3.55)	98.99 (2.86)
	All (N=15)	94.81 (7.93)	93.10 (13.35)	95.78 (14.35)

^a^SD: standard deviation.

## Discussion

### System Development

The tool described in this paper successfully automates the process of creating personalized IMU-based exercise technique classification systems. The previously laborious sequence of data collection, data labeling, and data analyses in software such as MATLAB (MathWorks, Natwick) has been streamlined as an Android tablet app that can be used by an exercise professional. The app eliminates the need for a data analysis professional to develop the classification systems by automating the common steps in the development of such systems (Figure 1). A key benefit of this tool for exercise professionals is that it allows rapid development of personalized exercise feedback systems tailored to their client’s exercise needs and specific movement patterns.

There are a number of notable benefits to taking an individualized analysis approach to the development of IMU-based exercise technique analysis systems. Recent work has shown such systems to be more accurate and computationally efficient than global classifiers [27]. The development of global classifiers is extremely time-intensive and requires hundreds of hours of data collection and analysis by researchers. Data must be collected in such fashion for any exercise for which a technique classifier is desired. This means that, currently, there exist only a handful of exercises that have been proven to be possible to assess with IMUs. The system described in this paper should allow for the creation of a personalized exercise classifier for any rehabilitation or S&C exercises that are cyclical and repetition based. Therefore, clinicians would not be limited in their exercise choices when designing specific programs to meet their clients’ needs. The app described in this paper could be conceivably used by a clinician during a patient’s visit to their clinic, and then the data labeled from this session could be used to create a functioning analysis tool for their program, which they may complete in the absence of professional supervision.

### System Evaluation

The preliminary evaluation of the system also suggests that the accuracy, sensitivity, and specificity of the personalized exercise technique classifiers may exceed that of global exercise technique classification systems. This reflects other similar research that compared sensor setups and classification methodologies for the barbell squat and deadlift exercises [27]. Although it is difficult to make direct comparisons with the previous research, it can be noted that a single IMU positioned on the left thigh has been demonstrated as capable of assessing acceptable or aberrant lunge technique with 77% accuracy [17] and single-leg squat technique with 75% accuracy [18]. These values were computed using leave-one-subject-out cross-validation. The personalized systems, evaluated in the real world, achieved 84% and 97% accuracy for the same analysis of lunges and single-leg squats, respectively. The binary classification of squat technique has previously been shown to be 80% accurate in a global classification system using a single lumbar-worn IMU [16]. The individualized systems described in this paper ranged from 50% to 100% accuracy and had a mean value of 85% across the 15 participants. It can also be noted that the deviations collected from the 5-participant beginner group used for analysis in this paper were naturally occurring, whereas in the aforementioned lunge and squat global classifiers, the deviations from correct technique were deliberately induced by study participants. This may make individualized classifiers more functional and usable in the real world. This paper’s deadlift accuracy result of 95% exceeds recently published work on binary classification of the deadlift with a left thigh IMU where 84% accuracy was achieved [27]. This is likely because there was more training data for each individual in this study. The personalized classification systems used in this preliminary evaluation of the tablet app were developed using 4 sets of each exercise (a total of 40 repetitions). Increasing the amount of training data used for each individual would likely further improve the accuracy of their personalized exercise technique evaluation system [24,25].

### Limitations

There are a number of contextual factors to this study that should be considered. Most notably, although the tool described allows for the efficient creation of an IMU-based exercise technique classifier for any cyclical, repetition-based exercise, it is not as simple as using a global classification system for exercises for which they exist. The tool described requires at least one recorded session with an exercise professional and requires the exercise professional’s time and expertise to label the video data. However, the tool described could be conceivably used to fill in the gaps in a client’s exercise program where a global classifier is not yet available. Moreover, the labeled data can all be stored in a database, and the data that were initially used to create individualized classifiers can be pooled together to make a global classifier. The exercise professional could switch to this global classifier when they deem it accurate enough to negate the benefits of creating an individualized classifier for each of their clients.

A key area that limits the findings of the evaluation study is that it was small scale, and the participants were not balanced in experience or gender. Moreover, the study participants were relatively homogenous in the evaluation study, and it is not yet understood whether the results found would be generalizable to other populations such as older, obese, or underweight people. In particular, the system evaluation was completed with individuals not currently undergoing rehabilitation. Future work should investigate the system with individuals undergoing rehabilitation. It is foreseen that it should still work, provided the exercise professional can label the data appropriately for each individual’s needs. The authors also acknowledge that more work is required to assess the capabilities of classifiers created with this new tool, particularly in the detection of exact deviations in exercise technique. The capabilities of a multiple IMU setup must be examined. However, the results presented show excellent potential for a single IMU setup to assess complex compound lower limb exercises when using personalized classifiers.

### Future Work

It should be noted that this paper only describes the development of this new tool and its first evaluation. It is not yet fully understood how it will be incorporated into clinical practice. Future work should investigate the influence of the exercise professional’ s experience level, when labeling the data, on system accuracy. The usability of the system and how it may best be incorporated into a clinician’s use of time should also be investigated. Only 1 exercise professional labeled the data in the evaluation study. The coding was not compared with other professionals; this should be investigated in future studies. Finally, the tool described only replicates current state of the art in the field, and the signal processing, feature computation, and classification methods ought to be iterated as the field progresses.

### Conclusions

In this paper, a tablet app that streamlines the creation of IMU-based exercise technique analysis systems is presented. The tool replicates the data analysis pathways that have been used in recently published research [16-19]. It also allows an exercise professional to record video data simultaneously to IMU data and label it efficiently, following a session with a client. The app then creates personalized exercise technique classifiers for the client based on the labeled IMU data. These personalized classifiers are less memory-intensive and more accurate than equivalent global classifiers for the exercises used in this study. In addition to this, data collected with the tool could ultimately be used to train new global classification systems with increased accuracy because of the increased amount of training data available.

## Abbreviations

FN: false negative
FP: false positive
IMU: inertial measurement unit
S&C: strength and conditioning
SD: standard deviation
3D: three-dimensional
TN: true negative
TP: true positive
